# Dietary Strategies for Management of Metabolic Syndrome: Role of Gut Microbiota Metabolites

**DOI:** 10.3390/nu13051389

**Published:** 2021-04-21

**Authors:** Sonia Croci, Lina Ilaras D’Apolito, Valeria Gasperi, Maria Valeria Catani, Isabella Savini

**Affiliations:** Department of Experimental Medicine, Tor Vergata University of Rome, 00133 Rome, Italy; Sonia.croci@gmail.com (S.C.); Dottoressadapolito@gmail.com (L.I.D.); savini@uniroma2.it (I.S.)

**Keywords:** metabolic syndrome, gut microbiota, dysbiosis, nutrition, inflammation, probiotics, prebiotics

## Abstract

Metabolic syndrome (MetS) is a complex pathophysiological state with incidence similar to that of a global epidemic and represents a risk factor for the onset of chronic non-communicable degenerative diseases (NCDDs), including cardiovascular disease (CVD), type 2 diabetes mellitus, chronic kidney disease, and some types of cancer. A plethora of literature data suggest the potential role of gut microbiota in interfering with the host metabolism, thus influencing several MetS risk factors. Perturbation of the gut microbiota’s composition and activity, a condition known as dysbiosis, is involved in the etiopathogenesis of multiple chronic diseases. Recent studies have shown that some micro-organism-derived metabolites (including trimethylamine N-oxide (TMAO), lipopolysaccharide (LPS) of Gram-negative bacteria, indoxyl sulfate and p-cresol sulfate) induce subclinical inflammatory processes involved in MetS. Gut microbiota’s taxonomic species or abundance are modified by many factors, including diet, lifestyle and medications. The main purpose of this review is to highlight the correlation between different dietary strategies and changes in gut microbiota metabolites. We mainly focus on the validity/inadequacy of specific dietary patterns to reduce inflammatory processes, including leaky gut and subsequent endotoxemia. We also describe the chance of probiotic supplementation to interact with the immune system and limit negative consequences associated with MetS.

## 1. Introduction

Metabolic syndrome (MetS) is defined by WHO as a pathological condition characterized by obesity, insulin resistance, hypertension, hyperlipidemia and waist-to-hip ratio; MetS is present if three or more of the above-mentioned criteria are present [1].

Main MetS risk factors are a sedentary lifestyle and high sugar or fat diets [1], which over time lead to being overweight and obesity. An excess of adipose tissue, particularly in the visceral compartment, is associated with morbidity and complications of MetS, because of the increased production of inflammatory molecules (cytokines and adipokines) [2,3]. Adipose tissue is, indeed, an active and complex endocrine organ that secretes molecules, playing a key role in inflammation, immune response, appetite regulation, vascular events, reproductive functions, and insulin sensitivity [4].

Recent scientific data have linked MetS development also to intestinal dysbiosis [5,6]: because of increased intestinal permeability, the translocation of lipopolysaccharide (LPS), an outer membrane component of Gram-negative bacteria, occurs, thus determining metabolic endotoxemia, which can be viewed as a causal factor of chronic low-grade systemic inflammation [7,8].

The complex ecosystem of microorganisms (including bacteria, viruses, protozoa, and fungi) living in different districts of the human body (gastrointestinal tract, skin, mouth, respiratory and urogenital systems) is defined as microbiota. Most microbiotas reside in the gastrointestinal tube [9]. The microbiota contains over 100 times more unique genes than those codified in the human genome [10]: it encompasses over 100 trillion microbes and 5000 different species, accounting for 5 million genes.

Human microbiotas vary according to diet, lifestyle and the presence or absence of specific diseases. Indeed, it has been shown that a high fat and sugar diet alters the intestinal ecosystem (at the phyla, genus, and/or species level), triggering changes in metabolic pathways and inducing pro-inflammatory cascade signals [11,12]. Some studies have shown that dysbiosis causes low-grade inflammation, obesity and consequently MetS [13], while others have shown that dysbiosis is the result of low-grade inflammation during obesity and MetS [2,14]. Further investigations are needed to understand if dysbiosis is a cause or consequence of MetS. Nonetheless, modulation of dysbiosis via dietary interventions and probiotic supplementation has provided evidence for supporting a possible beneficial effect in treating MetS complications [15].

There is growing evidence about the role of bacterial metabolites as pathophysiological biomarkers of cardio-metabolic diseases, with applications in diagnostics and prognosis [16]; for example, plasma levels of trimethylamine N-oxide (TMAO; derived from choline and carnitine) are associated with various diseases [17,18], while indoxyl sulfate (derived from tryptophan breakdown) and p-cresyl sulfate (derived from tyrosine and phenylalanine), as well as their metabolites [19], are involved in the progression of kidney and vascular diseases (Table 1) [20].

The habitual diet plays an important role in defining the composition of the intestinal microbiota and determining the microbial metabolites that can affect the host metabolism. In the literature, several studies have shown positive effects of some dietary models in MetS management. For example, adherence to the Mediterranean diet (MD) leads to significantly higher levels of total short-chain fatty acids (SCFAs), important gut microbiota metabolites that modulate immune–endocrine processes [44].

The purpose of this review is to examine and evaluate potential microbial metabolites involved in MetS to define nutritional interventions aimed at modulating the intestinal microbiota and personalizing MetS treatment.

## 2. SCFA Beneficial Metabolic Effects

Most bacteria found in the gastrointestinal tract belong to Firmicutes (Lactobacillus, Blautia, Clostridium and Enterococcus genera) and Bacteroidetes (Bacteroides, Parabacteroides, Porphyromonas, Prevotellaceae (Prevotella) and Rikenellaceae (Alistipes) genera) phyla, followed by Actinobacteria (Bifidobacteria), Proteobacteria (Escherichia coli), Fusobacteria, Verrucomicrobia and Cyanobacteria phyla [45,46,47].

**Figure 1:**
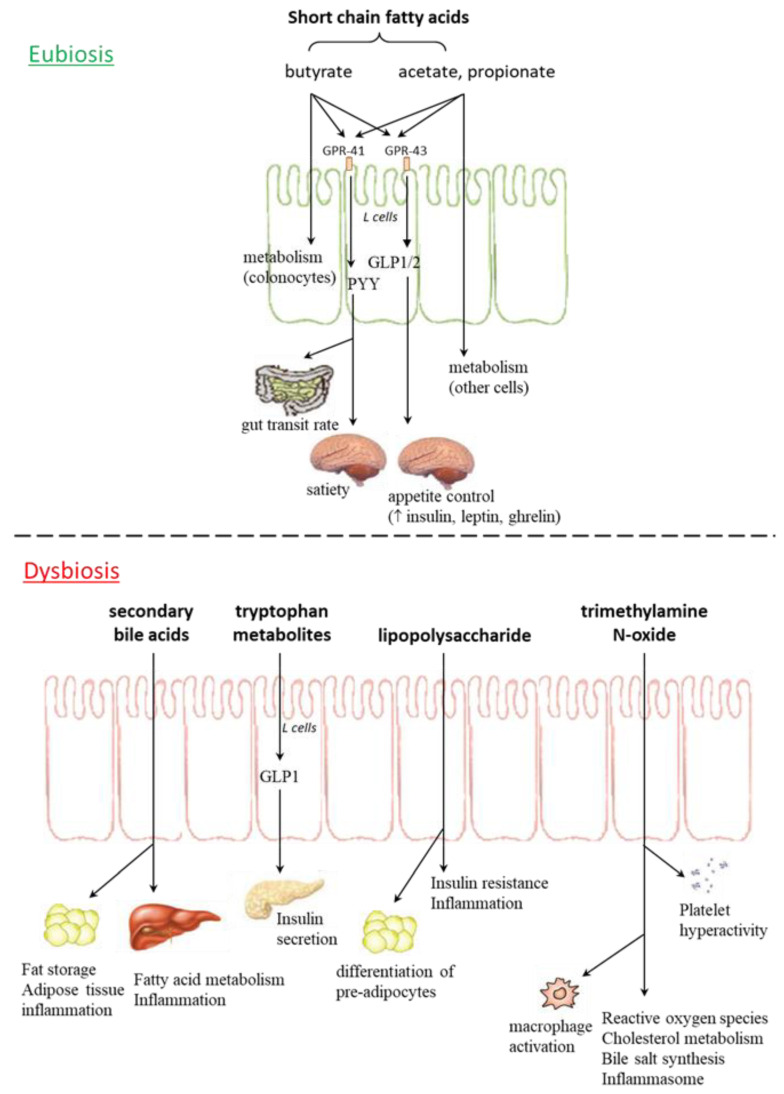
Gut microbiota metabolites in eubiosis and dysbiosis. GPR: G protein coupled receptor; GLP: glucagon-like peptide; PYY: peptide YY.

Intestinal bacteria play an important role in regulation of the host metabolism (influencing energy homeostasis, appetite and food eating behavior) and modulation of the immune system, through the production of SCFAs, vitamins, metabolites, and neuropeptides (Figure 1) [48,49].

Firmicutes bacteria produce SCFAs as products of the hydrolysis and fermentation of dietary polysaccharides; once taken up by enterocytes, butyrate is usually used as an energy source for intestinal epithelium, while acetate and propionate enter in systemic circulation and reach peripheral tissues, where they are used for lipogenesis and gluconeogenesis.

SCFAs also act as signaling molecules, thereby exerting beneficial metabolic effects, including the regulation of intestinal transit time [21]; modulation of food intake [50,51]; increase in intestinal absorption of monosaccharides (by inducing the expression of sodium/glucose transporter-1) [50]; increase in lipogenesis by inhibiting the inhibitor of lipoprotein lipase in the small intestine (thus inhibiting fatty acid release from triglycerides) and promoting cellular uptake of triglycerides (thus stimulating their storage in depot organs) [52].

Specifically, SCFAs stimulate colonic L cells through activation of the G protein-coupled receptors GPR41 (Free Fatty Acid Receptor 3 or FFA3) and GPR43 (Free Fatty Acid Receptor 2 or FFA2); the first receptor triggers the release of the anorexigenic peptide PYY, which increases the gut transit rate and satiety, while the second one reduces inflammation, stimulates release of glucagon-like peptide 1 and 2 (GLP1 and GLP2) and increases concentrations of peripheral hormones, such as insulin, leptin and ghrelin, thus influencing appetite control [51].

## 3. LPS and Endotoxemia

In healthy conditions, the integrity of the intestinal epithelial barrier, guaranteed by a tight junction network, blocks passage of antigens or microbe-derived endotoxins. Some pathological conditions give rise to gut microbiota perturbations (referred to as dysbiosis) and a subsequent impairment of intestinal barrier function (due to disorganized tight junction proteins, zonulin and occludin, in colonocytes); in these circumstances, microbial metabolites can cross the intestinal barrier and move to the bloodstream, triggering systemic pro-inflammatory signaling that, in turn, causes metabolic alterations (peripheral insulin resistance, hyperglycemia and non-alcoholic fatty liver disease (NAFLD)) in distant tissues [11,53]. Microbe-derived pro-inflammatory molecules include LPS, flagellins and peptidoglycans, which can reach the bloodstream, leading to “metabolic endotoxemia” [54]; this condition causes or exacerbates human pathologies, from intestinal (inflammatory bowel diseases, colon cancer) to neurological ones (Parkinson’s disease, autism), including MetS, obesity, multiple organ failure, renal and traumatic brain injuries, autoimmunity, transplant rejection, depression, chronic fatigue, and HIV disease [23,55,56].

Obesity, insulin resistance, and NAFLD, linked to MetS, are usually associated with low diversity in the gut microbiome and chronically higher levels of pro-inflammatory and microbiota derived LPS in circulation [57]. A growing body of evidence suggests the potential role of LPS in obesity, insulin resistance, hepatic steatosis, and systemic and local inflammatory processes [11]. The ability of LPS to induce proliferation and adipogenesis is supported by in vitro and in vivo (mice models and human subjects) studies. LPS has been shown to promote differentiation of pre-adipocytes in culture, via Janus kinase/signal transducers and activators of transcription (JAK/STAT) signaling and AMPK-regulated cytosolic phospholipases A2 (cPLA2) expression [58]. A low dose (300 μg/kg/day) LPS injection in lean mice grown with a normal chow diet leads to weight gain, adipose tissue stores, metabolic alterations, and increased expression of pro-inflammatory markers [54,59,60]. In human studies, *E. coli* LPS exposure (3 ng/kg) has been shown to promote systemic insulin resistance and adipose tissue-related inflammation [61]. Specifically, low dose (0.6 ng/kg) intravenous LPS administration induces a rapid, transient increase in plasma interleukin (IL)-6 (25-fold) and tumor necrosis factor α (TNFα; 100-fold), followed by modest induction of pro-inflammatory (i.e., IL-6, TNFα, monocyte chemoattractant protein-1 (MCP-1), suppressor of cytokine signaling 1 and 3 (SOCS1 and SOCS3)) cytokine expression in adipose tissue [62]. In addition, whole body insulin sensitivity and homeostatic model assessment for insulin resistance (HOMA-IR) are significantly impaired after 24 h of an LPS injection [61]. However, a weakness of these studies is that the immune response can be stimulated by an administered circulating dose of LPS greater than that actually released by bacteria; there are no studies capable of quantifying bacterial LPS production.

In vitro molecular studies show that LPS impairs insulin sensitivity through activation of Toll-like receptors (TLRs): in particular, LPS binds to LPS-binding protein, activates the CD14 receptor and transfers TLR4 in the plasma membrane of macrophages [61]. In macrophages and dendritic cells, LPS also regulates nucleotide oligomerization domain (NOD)-like receptors, thus triggering activation of pro-inflammatory transcription factors, including nuclear factor-κB (NF-κB), activator protein-1 (AP-1) and interferon regulatory factors (IRFs); among downstream targets, several components of the inflammasome (caspase-1 and apoptosis-associated speck-like protein containing a caspase recruitment domain (ASC)) are transcribed [63,64,65,66]. Collectively, these events regulate glucose and lipid homeostasis; accordingly, Cani and colleagues [54] reported increased expression of pro-inflammatory (TNFα, IL6, IL-1), oxidative stress (NADPH oxidase, inducible nitric oxide synthase (iNOS)) and macrophage infiltration (CD86) markers in the liver of genetically obese mice with metabolic endotoxemia [54,59,60]. Conversely, a systematic review, analyzing serum concentrations of LPS and/or LPS-binding protein in diabetic patients, highlighted that high LPS levels were observed both in diabetic subjects and healthy controls [67]. In order to study the actual involvement of LPS in glycemic regulation, these human studies require further confirmation, conducted on larger and uniform samples (for age, diet, lifestyle, cigarette smoking, use of drugs, etc.). In addition, it would be interesting to investigate if, in diabetic subjects, inflammatory markers are really correlated with LPS serum levels.

## 4. Nonalcoholic Fatty Liver Disease (NAFLD) and Microbiome

NAFLD is the hepatic manifestation of cardiometabolic syndrome. Systemic LPS concentration is significantly elevated in NAFLD compared to control groups, in both human and animal studies [68,69,70,71]. The gut microbiota contributes to liver fat deposition through modulation of the nuclear farnesoid X receptor (FXR), responsible for regulation of bile acid synthesis, and hepatic triglyceride accumulation [8,11,55]. After a meal, primary bile acids (chenodeoxycholic and cholic acids), stored in the gall bladder, are secreted in the duodenum, where they can be deconjugated by gut microbes, thus being metabolized into secondary bile acids in the colon [72]. Bacteria with the capability of producing secondary bile acids belong to *Clostridium* (clusters XIVa and XI) and *Eubacterium* [73,74].

Gut microbiota involvement in NAFLD genesis has been demonstrated by microbiota transplantation in recipient germ-free mice, as it generates fasting hyperglycemia, insulinemia and NAFLD; in particular, *Lachnospiraceae bacterium 609* and *Barnesiella intestinihominis* species were specifically related to NAFLD [75]. The gut microbiota contributes to NAFLD development also by increasing the number of ethanol-producing bacteria (e.g., *Escherichia coli*, *Klebsiella pneumoniae*), thereby favoring gut permeability alteration, generation of reactive oxygen species (ROS), and liver inflammation [76].

However, it should be taken into account that the genetic background of the mice (wild-type, inbred strains and their transgenic derivatives) strongly affects gut microbiota composition [55] and, moreover, each mammalian species harbors a distinct microbial composition [15], thus limiting the translation of results obtained in animal models to humans.

## 5. Role of Bacterial TMAO and Development of Atherosclerosis

**Figure 2:**
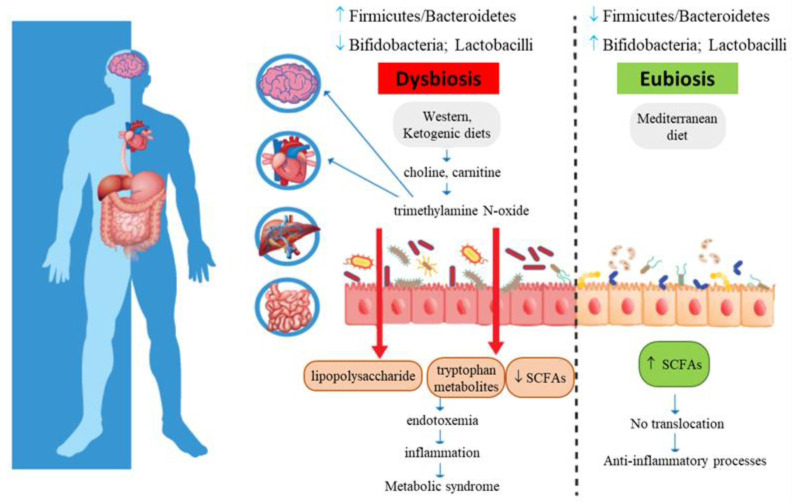
Metabolic syndrome and gut microbiota intestinal dysbiosis. Increased intestinal permeability causes translocation of lipopolysaccharide and tryptophan-derived metabolites, with subsequent metabolic endotoxemia and chronic low-grade systemic inflammation.

TMAO is a biomarker of risk for major adverse cardiovascular and cerebrovascular events, such as myocardial infarction and stroke (Figure 2): increased plasma TMAO concentrations have indeed been correlated with the accumulation of fatty depots in blood vessels, fatty liver, visceral obesity, and atherosclerosis [29,30,77,78,79,80,81,82,83].

Lecithin (the phospholipid phosphatidylcholine, found in eggs, liver, red meat, and fish) is the major dietary source of choline, a semi-essential nutrient part of the B-complex vitamin family [84]. Choline takes part in lipid metabolism, as well as in the synthesis of acetylcholine, homocysteine, and methionine [31]. Choline can be metabolized by gut microbiotas (mainly Enterobacteriaceae), with the production of trimethylamine (TMA), dimethylamine (DMA) and monomethylamine (MMA), which reach the liver where they are converted into TMAO via hepatic flavin-monooxygenase-3 [32]. Therefore, dietary consumption of choline may lead to the generation of nitrosamine precursors with carcinogenic activity; it is noteworthy that TMA-producing bacterial strains (such as *Escherichia fergusonii*, *Clostridium sporogenes*, and *Proteus penneri*) have been detected in the human gut [31,85].

High TMAO serum levels are related to MetS and cardiovascular risk. In 4007 healthy participants, deuterium [D9]-labeled phosphatidylcholine and the ingestion of two hard-boiled eggs have been shown to increase plasma TMAO concentrations that are reduced to baseline levels by oral broad-spectrum antibiotics [33]. The same group demonstrated a direct relationship between the incidence of major adverse cardiovascular events (death, myocardial infarction, or stroke) and TMAO plasma levels, in a long-term study (three years of follow up) [33]. Similarly, in a cohort of 339 patients, plasma TMAO concentrations were higher in patients with diabetes compared to euglycemic subjects, as well as in patients with MetS compared to healthy individuals; also, a direct relationship between plasma TMAO levels and cardiovascular adverse events has been reported [34]. In 220 subjects, fasting serum TMAO levels positively correlate with age, body mass index (BMI), fasting glycemia, blood lipids and carotid intima-media thickness [29]. A meta-analysis by Schiattarella et al. demonstrated a positive dose-dependent association between circulating TMAO levels and all causes of mortality for cardio/cerebrovascular events [35]. A cross-sectional observational study, carried out on 330 adult subjects with MetS, documented that circulating TMAO levels positively increased with BMI, as well as with MetS indexes (such as the visceral adiposity index and the fatty liver index) [36]. Additionally, a gender-specific relationship between TMAO levels and MetS markers (obesity, blood pressure, serum lipids, serum glucose and insulin resistance-related index) was identified in 1081 subjects [26]. Finally, relationships between the gut microbiota composition and circulating metabolites, including fasting plasma TMAO levels, have been identified in 531 middle-aged Finnish men, enrolled in the METabolic Syndrome In Men (METSIM) study: specifically, (i) the most dominant species belong to Bacteoridaceae, Ruminococcaceae and Lachnospiraceae families; (ii) the abundance of Peptococcaceae and *Prevotella* appears to be strictly linked to TMAO levels; (iii) there is a negative association between TMAO concentrations and an abundance of *Faecalibacterium prausnitzii* [7].

What are the molecular pathways by which TMAO exerts its pathogenic effects? TMAO may promote dyslipidemia by regulating hepatic lipogenesis and gluconeogenesis [10], macrophage scavenger receptors [27], while downregulating cholesterol and bile acid metabolism [28], as well as impairing macrophage reverse cholesterol transport [86], promoting movement of activated leukocytes to endothelial cells [18], activating NF-κB signaling [18], and enhancing platelet activation, thus promoting a pro-thrombotic phenotype [17] and inducing endothelial dysfunction through activation of the NLRP3 inflammasome [87]. In addition, TMAO also affects brain functions, as it induces neuronal senescence, increases oxidative stress, impairs mitochondrial function, inhibits mTOR signaling and upregulates expression of macrophage scavenger receptors and CD68, all phenomena that contribute to brain aging and cognitive impairment [18,83,88]. These studies suggest that analysis of serum or cerebrospinal fluid TMAO levels could represent, in clinical practice, a novel prevention and treatment tool.

## 6. Gut Microbiota and Tryptophan Metabolism

Tryptophan (Trp) is an essential aromatic amino acid that is present in oats, bananas, dried prunes, milk, tuna fish, cheese, bread, poultry, peanuts, and chocolate. Gut microbiota can directly utilize Trp (approximately 4–6%), limiting its bioavailability [37]. Bacteria-derived indoles, produced from Trp metabolism, can modulate host physiological and pathological pathways, thus contributing to cardiovascular, metabolic, and brain disorders [38]. For example, *Clostridium sporogenes* produces indole propionic acid from dietary Trp that plays an important role in the integrity of the intestinal barrier.

Trp metabolites are also involved in MetS. Indeed, in human patients with MetS, overactivation of indoleamine 2,3-dioxygenase and increased serum levels of kynurenine have been reported [89]. Indoxyl sulfate and p-cresyl sulfate are two other Trp metabolites that stimulate GLP-1 in L cells and subsequent insulin secretion from pancreatic β cells [39,90]. These two metabolites seem to be also related to chronic kidney disease, a MetS complication, and related risk factors (cardiovascular disease (CVD); hypertension, diabetes and hyperhomocysteinemia) [40,41]; however, studies are few and sometimes controversial [42,91], so more investigations will certainly be useful for identifying future MetS and chronic kidney disease diagnostic markers.

## 7. Different Dietary Patterns and Microbiota Enterotypes

Diet strongly affects gut microbiota composition. Comparison of the fecal microbiota of European children and of children from a rural African village of Burkina Faso (following a polysaccharide-rich diet), for example, revealed significant differences: in Burkina Faso children, the predominant bacteria were Bacteroidetes, mainly *Prevotella* and *Xylanibacter* genera, while European children had significantly less SCFAs and more Enterobacteriaceae (*Shigella* and *Escherichia*) [22]. Diet-based differences have been confirmed in other studies: animal-based diets decrease levels of Firmicutes that metabolize plant polysaccharides (*Roseburia*, *Eubacterium rectale* and *Ruminococcus bromii*), while increasing the abundance of bile-tolerant microorganisms (*Alistipes*, *Bilophila* and *Bacteroides*) [92]. Accordingly, Prevotellaceae are prevalent in vegetarians, whereas the microbiotas of omnivores includes taxa Clostridiaceae, Bacteroidales and *Eubacterium* [20,93,94,95,96].

The gut microbiome is able to respond to short-term macronutrient changes; volunteers placed on either plant-(grains, legumes, fruits, and vegetables) or animal-(meats, eggs, and cheeses) based diets for five days showed significant temporal alterations in their microbial communities [92]. In contrast, such modulation determined by short term dietary intervention has not been found in Wu’s investigation, highlighting that, for relevant composition changes, long-term dietary patterns are needed: indeed, in fecal samples from 98 individuals, changes in gut microbiota composition occur rapidly (within 24 h) when shifting to a new dietary regimen, but only after 10 days, samples could be specifically divided into three enterotypes (mainly *Bacteroides*, associated with animal proteins/saturated fats, and *Prevotella*, associated with carbohydrates) [20]. Nevertheless, large cohort studies demonstrated that enterotypes might not always be stable and that enterotype stratification is not so robust among populations [97,98]. Further studies (especially concerning enterotype analysis in homogenous samples and long dietary interventions) are needed in order to define stable microbiota categorizations, although different endogenous and exogenous variables cannot always be controlled.

## 8. Dietary Strategies for MetS Management and Gut Microbiota Modulation

In MetS patients, nutritional intervention should firstly aim at reducing CVD and type 2 diabetes risk and usually includes reduction of body weight by 7–10%, followed by weight maintenance and lifestyle changes (such as increases in physical activity and stopping cigarette smoking). Losing as little as 5% of initial weight results in insulin sensitivity improvement, serum triglycerides and LDL-cholesterol reduction and decrease in systolic and diastolic blood pressure [99,100].

Obesity is one of the five fundamental features of MetS; the relationship between obesity and microbes is clearly established. Colonization of germ-free mice with gut microbiotas derived from obese subjects led to increases in total weight, compared with transplantation of lean gut microbiotas [101]. Similar results have been observed in humans, where obesity developed after fecal microbiota transplantation from overweight donors [102], whereas transplantation of lean microbiotas into individuals with MetS improved insulin sensitivity [103].

In obese subjects, main microbiota changes concern the reduction of Bacteroidetes and a proportional increase in Firmicutes and Actinobacteria [104,105,106,107]; increase in Bacteroidetes in overweight subjects has also been reported [108]. However, conflicting data still exist [105,109,110], especially considering differences in age, sex, physiological state, and ethnicity; therefore, we are far away from understanding the real Firmicutes/Bacteroidetes ratio in obesity, and further studies are recommended.

The Western diet, rich in saturated and trans fatty acids as well as sucrose intake, and low in fiber from fruits and vegetables, is linked to insulin resistance, dysbiosis and chronic non-infectious degenerative diseases [111,112,113,114]. The Western diet decreases microbial richness and increases the Firmicutes/Bacteroidetes ratio, in addition to increasing pro-inflammatory bacteria, and triggering changes at family, genus and species levels [20,22,24,25]. Conversely, a diet rich in ω-3 polyunsaturated fatty acids (PUFAs) is generally associated with anti-inflammatory effects [115]. Human studies on ω-3 PUFAs and gut microbiotas are based on PUFA supplementation or eicosapentaenoic (EPA) and docosahexaenoic (DHA) acids-rich drinks; nonetheless, these studies highlighted the PUFA ability to increase the abundance of SCFA-producing bacterial genera [116]. Among butyrate-producing bacteria, the following genera, belonging to the Lachnospiraceae family of the phylum Firmicutes, have been found: *Eubacterium*, *Roseburia*, *Anaerostipes*, and *Coprococcus* [117,118]. Mokkala et al. [119] showed that ω-3 PUFAs are associated with intestinal permeability in vivo and linked to concentrations of serum zonulin (a marker of intestinal permeability), but the study was performed only in overweight pregnant women.

Several in vivo studies have underlined the relevance of dietary fat quality on metabolic health and gut microbiota composition. Patterson and co-workers [120] demonstrated that mice fed with high-fat diets of different compositions displayed peculiar microbial ecosystems: dietary saturated fatty acids (palm oil) were associated with a low abundance of Bacteroidetes, obesity and MetS; mono-unsaturated fatty acids (MUFAs; olive oil) led to an increase in the Bacteroidaceae family; ω-3 PUFAs (flaxseed/fish oil) increased EPA and DHA concentrations, as well as the intestinal abundance of *Bifidobacterium* genus. Accordingly, fatty acid profiles, by modulating the microbiota composition and integrity of the epithelial barrier, may contribute to low-intensity inflammation and, consequently, endotoxemia [24,121]. Similarly, in humans, ω-3 PUFAs have been shown to modulate the gut microbial community (increasing the Bacteroidetes/Firmicutes ratio) with positive metabolic outcomes on BMI, weight gain, obesity, insulin resistance, type 2 diabetes mellitus, and inflammatory bowel diseases [118,122,123,124,125,126,127]. MUFAs were also proven to positively impact the Bacteroidetes/Firmicutes ratio in 25 volunteers with risk of MetS: three MUFA-rich diets, indeed, increased *Parabacteroides*, *Prevotella*, *Turicibacter*, and Enterobacteriaceae’s populations, and changes in gut microbiota profiles were more evident in obese subjects [124].

MD adherence reduces chronic inflammation, improves lipid profile, insulin sensitivity and endothelial function, while decreasing CVD incidence and all causes of mortality (myocardial infarction, stroke, cancer) [128,129]. MD is characterized by high intakes of vegetables, legumes, fruits, nuts, grains, fish, seafood, and poultry as sources of proteins, and low intakes of dairy products, red and processed meat, cream, and sugar drinks; lipid sources mainly include foods rich in unsaturated fatty acids, as well as other beneficial active compounds (e.g., olive oil, fish, and nuts) [130]. Recently, the focus has shifted to the anti-inflammatory properties of polyphenols, as the protective roles of coffee and tea consumption against MetS have been proven for a long time [131]. Polyphenolic anthocyanins (e.g., blueberry) can lower triglycerides and fasting plasma glucose, while ameliorating systolic blood pressure. Eight-week supplementation of 4 cups of a freeze-dried strawberry beverage in MetS patients improved atherosclerosis risk factors by decreasing total and LDL-cholesterol levels and vascular cell adhesion-1 (VCAM1) expression [132].

High adherence to MD positively impacts the gut microbiota composition and microbial metabolomes. Individuals with low adherence show high urinary TMAO levels, while compliance to MD is linked to increased SCFA levels, and an abundance of *Prevotella* and fiber-degrading Firmicutes [133,134]. Likewise, an increase in Bacteroidetes and Clostridia, a decrease in species belonging to Protectobacteria and Bacillaceae, and a drop in TMAO levels, in following MD, have been reported [135]. Haro’s group [136] analyzed the ability of two dietary patterns (MD and low-fat diet) to resolve dysbiosis: 106 male subjects (33 obese patients with MetS, 32 obese individuals without metabolic complications and 41 non-obese controls), enrolled in the CORDIOPREV study, were subjected to the two different dietary interventions for 2 years. MD provided a minimum of 35% fats (22% MUFAs, 6% PUFAs and <10% saturated fats), 15% proteins and no more than 50% carbohydrates; the low-fat diet provided <30% total fats (<10% saturated fats, 12–14% MUFAs and 6–8% PUFAs), 15% protein and a minimum of 55% carbohydrates. In both diets, the cholesterol content was adjusted to <300 mg/day. What emerged from this study was that dysbiosis, characterized by a reduction of bacteria with saccharolytic activity and a subsequent reduction in SCFA production, was more severe in obese patients with MetS and both dietary patterns were able to reverse dysbiosis by inducing increases in *Bacteroides*, *Prevotella* and *Faecalibacterium* genera. In addition, MD also increased *Roseburia* and *Ruminococcus* genera, suggesting the greater potential of MD to restore gut microbiota functionality, as compared to a low-fat diet [136]. These results suggest that chronic dietary intervention may restore gut dysbiosis in obese patients with coronary artery disease, depending on the degree of metabolic dysfunction; it would be interesting to investigate gender differences since the study enrolled only men.

Some findings indicated that nutritional ketosis, induced by very low carbohydrate ketogenic diets (VLCKD; <10% carbohydrates per day), allowed weight management and improved metabolic and inflammatory markers, including lipids, glycated hemoglobin, high-sensitivity C-reactive protein, fasting insulin and glucose levels [137,138]. A recent, small (only nine studies) meta-analysis underlined a contradictory role of VLCKD on the gut microbiome, as it decreased microbial α diversity and richness; in particular, this dietary pattern increased Enterobacteriaceae (potentially pathogenic and pro-inflammatory species) and decreased the abundance of beneficial *Bifidobacterium* species [139]. However, the meta-analysis included experimental studies conducted both in humans and murine models, and the human samples were not homogeneous in terms of age or pathological conditions.

A diet rich in proteins and low in carbohydrates promotes dysbiosis, causing an increase in pro-inflammatory bacteria and a reduction in SCFA-producing bacteria, with subsequent changes in bacterial metabolites; indeed, increased branched-chain fatty acids, indoxyl sulfate, p-cresol sulfate and TMAO have been observed with this dietary regimen [135,140,141].

Epidemiological evidence identifies the benefits of vegetarian dietary patterns (rich in fiber, but low in EPA and DHA) in both the prevention and treatment of MetS, CVD mortality and risk of coronary heart disease [142,143]. In adults, plant-based diets have beneficial effects on gut microbiota, increasing *Bifidobacterium* and *Lactobacillus* species, elevating SCFA-producing bacteria and promoting *Ruminococcus* enterotype. Accordingly, indoxyl sulfate and p-cresol sulfate mean excretion is approximately 60% lower in vegetarians than in omnivores [43]. In omnivores, proteins are of plant and/or animal origin, each type with unique digestibility and degradation patterns, depending on the microbes involved: colon bacteria involved in protein metabolism primarily include potentially pathogenic *Bacteroides*, Coliform, and *Clostridium*. Inhibition of these potential pathogens is often associated with the restoration of micro-ecosystem homeostasis by reducing the release of enterotoxins and adverse microbial metabolites [144].

## 9. Prebiotics, Probiotics, and Next Generation Microbes

Prebiotics are selectively fermented components leading to specific changes in the composition and/or activity of gut microorganisms that may be beneficial for the host’s health and wellbeing [145]. Among them, oligosaccharides, such as fructo-oligosaccharides (FOS) and galacto-oligosaccharides (GOS), represent complex carbohydrates with the most influence in *Bifidobacterium* and *Lactobacillus* growth [146]: 30 g per day of dietary fiber (soluble fraction) resulted in a higher abundance of *Bifidobacterium spp*. and *Lactobacillus spp.*, as well as elevated levels of fecal butyrate [147]. However, the beneficial effects are attributed to the simultaneous use of probiotics and prebiotics, according to the “synbiotic” concept formulated in 1995 by Gibson and Roberfroid [148]. The widely used and recently revised definition of prebiotic is now a substrate selectively utilized by host microorganisms, conferring health benefits [149].

Current probiotics include microorganisms proven to exert positive effects on human health, such as *Lactobacillus* and *Bifidobacterium* genera [150]. Most individuals use probiotics as preventive tools, and consumption of fermented foods (yogurt and kefir) may be inversely related to MetS [151,152], even if the available literature does not uniformly agree on effective gut mucosal colonization. Nonetheless, supplementation with specific strains of *Lactobacillus* and *Bifidobacterium* has been found to (i) improve epithelial and mucosal barrier functions, (ii) inhibit the growth of pathogenic enteric bacteria and reduce production of pathogenic toxins, (iii) mediate some of the negative consequences associated with the consumption of high-fat diets; (iv) modulate the immune system, blood glucose levels and lipid profiles [153].

Investigations on animal experimental models and humans suggested an important role of probiotics on MetS and its complications. In a mouse model of obesity, supplementation with *Lactobacillus rhamnosus* reduced visceral adiposity and diet-induced obesity, while improving the integrity of the gastrointestinal microbial lining [154]. In experimental atherosclerotic models (apolipoprotein E knockout mice feeding a high-fat diet for 12 weeks), supplementation with *Lactobacillus rhamnosus GG* changed a proportion of gut microbiotas and significantly reduced atherosclerotic plaque size [155]. *Lactobacillus reuteri V340* added to a hypocaloric diet, together with regular physical activity, has shown promising results for MetS management in animals and adult patients: supplementation once daily for 12 weeks, indeed, reduced IL-6 and soluble VCAM-1 [156]. In the same way, although based on limited sampling (only 25 individuals), consumption of milk containing *B. lactis HN019* reduced classical MetS parameters and related cardiovascular risk factors (BMI, total cholesterol and LDLs, pro-inflammatory cytokines) [157].

Specific commensal species, as novel probiotics (the so-called next-generation probiotics), exert positive effects by reducing inflammation and strengthening the epithelial barrier [158]. Among them, *Akkermansia municiphila* is a major propionate-producing bacterium, while *Eubacterium hallii*, *Eubacterium rectale*, *Roseburia inulinivorans*, *Faecalibacterium prausnitzii*, *Clostridium lavalense*, *Bacteroides uniformis* and *Ruminococcus bromii* are responsible for most butyrate production [159]. *Akkermansia muciniphila* (0.5–5% of total bacteria) is inversely associated with obesity, diabetes, cardiometabolic diseases, inflammatory bowel disease, hypertension, and liver diseases [160,161,162,163,164,165]. Amuc_1100, an outer membrane protein of *A. muciniphila*, is involved in gut epithelium integrity and is negatively associated with metabolic endotoxemia [6,165].

In overweight and obese volunteers, daily *Akkermansia muciniphila* supplementation, for 3 months, resulted in improved insulin sensitivity and reduced insulinemia and plasma total cholesterol [166]; accordingly, dietary interventions for reducing obesity (calorie restriction, energy reduction, diet rich in prebiotic fibers) are associated with increased levels of *A. muciniphila* [167]. These preliminary results suggest that *A. muciniphila* may be a promising new therapeutic agent for MetS, although safety and absence of side effects must be assessed.

Consumption of yogurt and other fermented products is positively associated with gastrointestinal and cardiovascular health, cancer risk, weight management, diabetes, and bone density [168]. Several studies evaluated the effects of kefir, a fermented milk product, on MetS. Kefir grains have a specific combination of bacteria (*Lactobacillus*, *Lactococcus*, *Streptococcus Leuconostoc*, and acetic acid bacteria) and yeasts (*Saccharomyces*, *Kluyveromyces*, and *Candida*), which exert anticarcinogenic, antimicrobial and anti-inflammatory activities [13,169]. In MetS patients, 12 week-consumption of kefir improved anthropometrical measurements, blood pressure, lipid profile, glycemic homeostasis (insulin and HOMA–IR) and inflammation (TNF–α and IFN–γ) [151]. In a prospective cohort study, fermented milk was associated with a 4% reduction in risk of stroke, ischemic heart disease, and cardiovascular mortality [152]. However, all described studies should be interpreted with caution because of the high heterogeneity and different probiotic strains (or different microbial content of kefir grains) used in these investigations.

## 10. Conclusions

A relationship between microbiota metabolites, endotoxiemia and MetS is clearly emerging. Recent findings have shown that some microorganism-derived metabolites (including TMAO, LPS, indoxyl sulfate and p-cresol sulfate) induce subclinical inflammatory processes involved in MetS and CVD [17,18,19,33,35,36]. Although many studies have been conducted in mice models, which display a different phylogenetic makeup of bacterial communities with respect to humans [170,171], nonetheless, some phenomena related to MetS are similar between animal and human studies, as is, for example, the link between endotoxemia and insulin resistance and diabetes [11,61,165,172].

Among factors (age, lifestyle, drugs, type of birth, cigarette smoking, etc.) influencing microbiota composition, dietary habits play a key role; much evidence underlines that some dietary patterns are more effective than others in modifying the gut community. In particular, MD, rich in beneficial nutrients and bioactive compounds, is particularly effective in modulating gut microbiota composition and inflammatory processes (including leaky gut and subsequent endotoxemia); compliance with MD is indeed linked to resolved dysbiosis, increased SCFA levels, reduced TMAO levels, and an abundance of *Prevotella* and fiber-degrading Firmicutes [133,135]. Contrastingly, the Western diet is low in “carbohydrates accessible by the microbiota” and leads to the extinction of specific bacterial strains, thus negatively affecting a series of metabolic functions. A vegetarian or high-fiber diet can reduce total choline intake, thereby modulating the composition of intestinal microbiotas and/or their metabolites, but these patterns do not provide an adequate intake of PUFAs, important for anti-inflammatory effects and integrity of intestinal epithelium. For these reasons, long term MD appears to be the best treatment for preserving the greatest richness in microbiota species.

Functional foods enriched with probiotic or synbiotic bacteria may also be useful in targeting microbiota composition or specific biochemical pathways, as they have been shown to significantly improve insulin sensitivity and the lipid profile [166]. Nevertheless, we are still far from bacterial therapy: individual diversity is remarkable because everyone has their own distinctive microbial pattern and, even if you try to modify it, the trend always seems to be restoration of the initial profile. For the same reason, data on fecal microbiota transplantation, clinically successful in the treatment of recurrent Clostridium difficile infections [173,174], are not solid and conclusive; the currently available studies are few [107,175,176] and with several study shortcomings, among which the most relevant are the lack of long-term follow-ups and of homogenous fecal samples from donors.

In conclusion, although promising, microbiotas and microbiota-derived metabolites may not actually represent diagnostic MetS markers and/or have real therapeutic power, since many limitations must be overcome. The future challenge is to fully understand how different dietary patterns may specifically modulate the composition of gut microbiota and whether these changes are long-lasting; only a strong and robust knowledge will allow stabilizing the microbial community toward a healthy phenotype and reducing the risk of MetS.

## Figures and Tables

**Table 1:** Gut microbiota metabolites, diet, and host effects.

Family	Metabolites	Diet	Effects	References
Prevotellaceae	SCFAs	Fiber-rich diet	Energy source for colonocytes Signaling molecules Regulation of intestinal transit time Modulation of host appetite and food intake	[21,22]
Enterobacteriaceae	LPS	Western diet	Metabolic endotoxemia Pro-inflammatory signaling	[11,23,24,25]
Enterobacteriaceae, Bacteoridaceae, Ruminococcaceae, Lachnospiraceae	TMAO	Dietary source of choline	Risk factor for MetS, cardiovascular and cerebrovascular events	[17,18,26,27,28,29,30,31,32,33,34,35,36]
Clostridiaceae	Indoxyl sulfate, p-cresyl sulfate	Western diet; VLCKD	Chronic kidney disease Cardiovascular, metabolic and brain disorders	[20,37,38,39,40,41,42,43]

LPS: lipopolysaccharide; MetS: metabolic syndrome; SCFA: short chain fatty acid; TMAO: trimethylamine N-oxide; VLCKD: very low carbohydrate ketogenic diet.
